# Olfactory deficit and gastrointestinal dysfunction precede motor abnormalities in alpha-Synuclein G51D knock-in mice

**DOI:** 10.1073/pnas.2406479121

**Published:** 2024-09-16

**Authors:** YoungDoo Kim, Joseph McInnes, Jiyoen Kim, Yan Hong Wei Liang, Surabi Veeraragavan, Alexandra Rae Garza, Benjamin David Webst Belfort, Benjamin Arenkiel, Rodney Samaco, Huda Yahya Zoghbi

**Affiliations:** ^a^Department of Molecular and Human Genetics, Baylor College of Medicine, Houston, TX 77030; ^b^Jan and Dan Duncan Neurological Research Institute at Texas Children’s Hospital, Houston, TX 77030; ^c^Department of Neuroscience, Baylor College of Medicine, Houston, TX 77030; ^d^Department of Pediatrics, Baylor College of Medicine, Houston, TX 77030; ^e^Department of Neurology, Baylor College of Medicine, Houston, TX 77030; ^f^HHMI, Baylor College of Medicine, Houston, TX 77030

**Keywords:** alpha-Synuclein, G51D mutants, Parkinson’s disease, olfactory deficit, gastrointestinal dysfunction

## Abstract

Parkinson’s disease (PD) is typically a sporadic late-onset disorder, which has made it difficult to model in mice. Several transgenic mouse models bearing mutations in *SNCA*, which encodes alpha-Synuclein (α-Syn), have been made, but these lines do not express *SNCA* in a physiologically accurate spatiotemporal pattern, which limits the ability of the mice to recapitulate the features of human PD. Here, we generated knock-in mice bearing the G51D *SNCA* mutation. After establishing that their motor symptoms begin at 9 mo of age, we then sought earlier pathologies. We assessed the phosphorylation at Serine 129 of α-Syn in different tissues and detected phospho-α-Syn in the olfactory bulb and enteric nervous system at 3 mo of age. Olfactory deficit and impaired gut transit followed at 6 mo, preceding motor symptoms. The *Snca^G51D^* mice thus parallel the progression of human PD and will enable us to study PD pathogenesis and test future therapies.

## Significance

Many Parkinson’s disease (PD) models overexpress α-Synuclein using heterologous promoters, which is adequate to demonstrate that excessive α-Synuclein is toxic but not ideal for learning the precise ontogeny of the disease pathogenesis, specifically where the disease starts and how it progresses. To answer these questions, it is beneficial to generate a mouse model expressing a disease-causing mutation under the endogenous promoter. Here, we generated three *Snca* knock-in mice. Among them, homozygous *Snca^G51D^* mice develop motor deficits by 9 mo of age. These mice exhibit olfactory and gastrointestinal abnormalities by 6 mo. They lose dopaminergic neurons and have reduced dopamine at 18 mo, respectively. Their spatiotemporal pattern of phosphorylated alpha-Synuclein and progression of symptoms parallel those of human PD.

Parkinson’s disease (PD) is the second most common neurodegenerative disease in the world (1). Classed as a movement disorder because of its hallmark tremors, rigidity, and bradykinesia, PD also causes a number of nonmotor symptoms. Hyposmia (reduced sense of smell), sleep disturbances, and slowed gastrointestinal transit can precede motor symptoms by 20 y (2–6). Although some of these early symptoms appear outside the central nervous system, they correlate with early alpha-Synuclein (α-Syn) pathologies in certain brain regions. Braak’s staging of PD progression indicates that during stages I and II, Lewy bodies—which consist largely of accumulated α-Syn—are formed in the olfactory tract as well as in the dorsal motor nucleus of the vagus nerve (DMV) that connects the brainstem to the intestine. This early accumulation of α-Syn affects both olfaction and intestinal motility (7, 8). As the disease progresses to Braak stages III and IV, the substantia nigra begins to show Lewy bodies accompanied by the development of motor symptoms (9).

Both sporadic and familial PD cases share the pathology hallmarks, such as accumulation of α-Syn. Autosomal dominant forms of PD, however, such as those caused by point mutations in Synuclein alpha (*SNCA,* which encodes α-Syn), or duplication or triplication of the gene locus, can cause much earlier onset (10–12). Of the six documented familial PD mutations in *SNCA* (A30P, E46K, H50Q, G51D, A53E, and A53T) (13–18), the G51D mutation causes the earliest onset, even in the 10 y, with an aggressive course that includes dementia and resembles multiple system atrophy (MSA), another Synucleinopathy (15, 17, 19). Several transgenic mouse models overexpress wild-type or mutant forms of α-Syn under brain-specific promoters or Bacterial artificial chromosome (20–24); other mouse models are generated by the injection of α-Syn-expressing virus (25) or preformed fibrils (PFF) of α-Syn (26) into the substantia nigra in mice. While these various mouse models have been useful to demonstrate the toxic effect of α-Syn in the substantia nigra, they do not reveal the typical course of PD due to the transgenic mice overexpression of α-Syn in brain regions that are typically unaffected in human PD (21). On the other hand, BAC-transgenic mice that express α-Synuclein properly in the brain do not show motor phenotypes (22). Finally, injection of α-Syn PFF or virus in the substantia or striatum prohibits assessment of pathogenesis in peripheral tissue (25, 26).

To achieve the correct spatiotemporal expression of mutant α-Syn and thereby better study the pathophysiological changes that occur in human PD patients, we developed three knock-in (KI) mouse models bearing PD-causing mutations in *Snca*. Here, we show that mice bearing the G51D mutation develop molecular pathologies and symptoms that parallel those observed in human PD patients. Most notably, the *Snca^G51D^* knock-in mice show early pathologic phosphorylation of α-Syn in the olfactory bulb and enteric nerve cells in the intestine, accompanied by impaired olfaction and gastrointestinal motility, well before showing motor symptoms. Taken together, these data suggest that disease pathogenesis starts from the olfactory bulb and enteric nervous system (ENS) and demonstrate that the *Snca^G51D^* KI line is a valuable mouse model that recapitulates the initial stages of α-Syn pathology in PD.

## Results

### Generation of *Snca* Mutant Knock-In Mice.

All six of the known inherited, disease-causing mutations of *SNCA* are located within the N-terminal region of the protein, which is highly conserved between humans and mice, except for the 53rd alanine (A) in humans, which is threonine (T) in mice, but a disease-causing mutation in humans (*SI Appendix*, Fig. S1*A*). In mice, we attempted to generate four human mutations (A30P, E46K, H50Q, and G51D), but H50Q mutation generation was not successful. Thus, we selected three mutants (A30P, E46K, and G51D) to model in mice by editing the endogenous murine *Snca* locus using the CRISPR-Cas9 system (27). We generated single-stranded oligodeoxynucleotides (ssODNs) containing target codon sequences for each mutatioMice Show Motor Incoordinationn with 75 to 100 base long homology arms for precise sequence replacement by homology-directed repair (*SI Appendix*, Table S1). We confirmed the correct substitution of the bases along with synonymous mutation (for genotyping purposes) using Sanger sequencing and genomic PCR of the targeted genomic regions (*SI Appendix*, Fig. S1 *B* and *C*). We observed that *Snca* messenger RNA (mRNA) and α-Syn protein levels in the KI mice were comparable to wildtype (WT) using qRT-PCR (*SI Appendix*, Fig. S1*D*) and western blot analysis (*SI Appendix*, Fig. S1*E*), with a relative increase in phosphorylation at S129 of α-Syn found in each KI mutant compared to WT (*SI Appendix*, Fig. S1 *E* and *F*).

### Homozygous *Snca*^*G51D*^ Mice Show Motor Incoordination and Gait Disturbance.

**Fig. 1.**
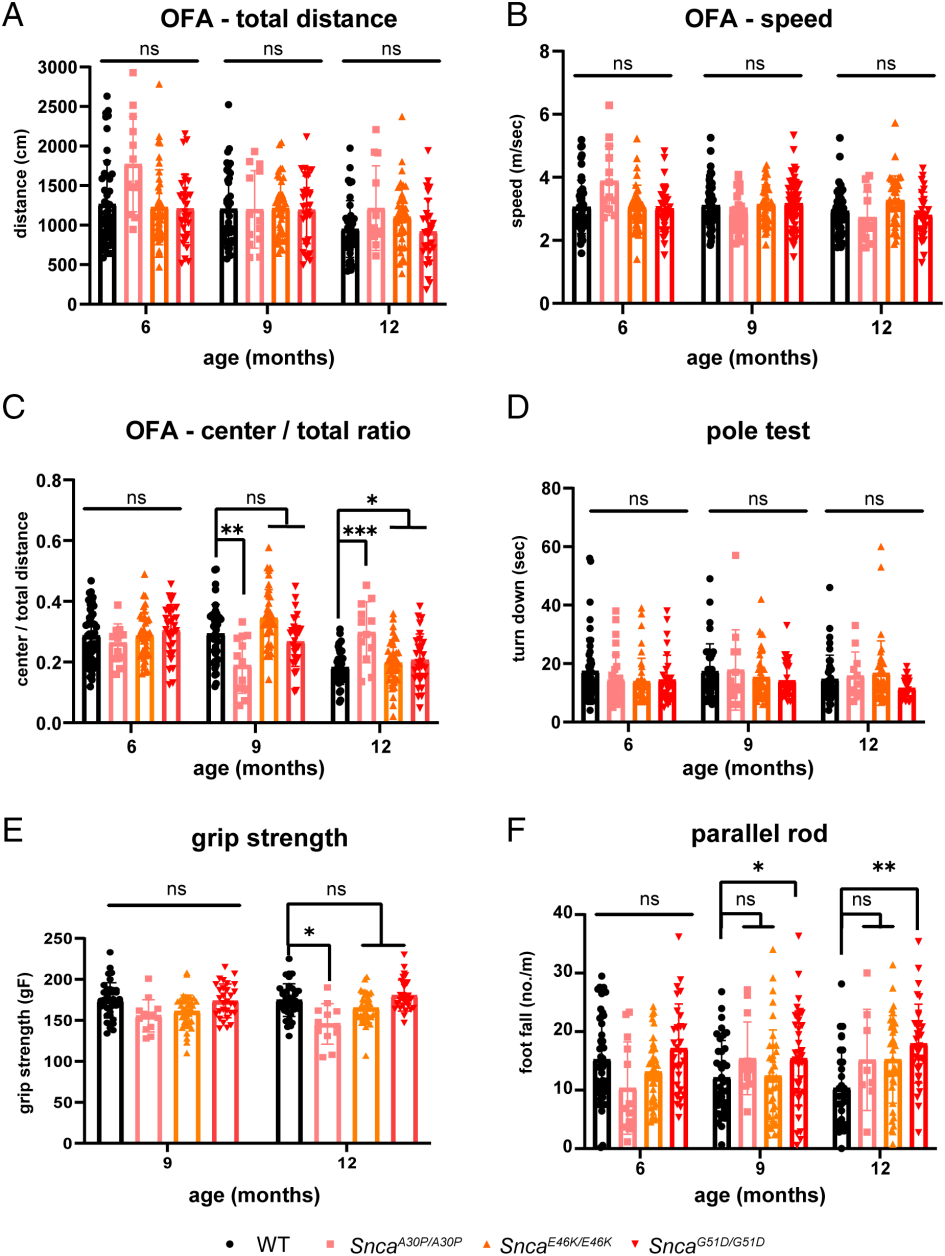
Characterization of the motor function of knock-in mice harboring one of three different α-Syn mutations. We compared motor function of all three mouse lines to wild-type littermates at 6, 9, and 12 mo of age. WT (n = 33: n = 17 male and n = 16 female), *Snca^A30P/A30P^* (n = 14: n = 7 male and n = 7 female), *Snca^E46K/E46K^* (n = 37: n = 18 male and n = 19 female), *Snca^G51D/G51D^* (n = 29: n = 15 male and n = 14 female). (*A*) Total distance traveled in the open-field test, (*B*) speed, and (*C*) movement in the center of the setup. (*D* and *E*) Motor strength was assessed by the pole-hanging test (*D*) and grip strength (*E*). (*F*) Motor coordination was measured by the number of footslips on the parallel rod floor test. *P < 0.05, **P < 0.01, ***P < 0.005, and n.s., not significant.

Because homozygosity for a mutant allele is often required to observe a phenotype during the short life span of mice (28, 29), we performed our studies on homozygous KI lines. Given that loss of fluidity, bradykinesia, stiffness, and balance problems are motor behaviors that are among the hallmarks of PD, we first assessed each KI mouse line for gross motor function. In the open-field test, none of the three KI mouse lines differed from WT in distance traveled or speed, at ages 6, 9, or 12 mo (Fig. 1 *A* and *B*). There was a mild but significant increase in central/total distance ratio at 9 (A30P KI) and 12 mo (all three KI) of age (Fig. 1*C*).

**Fig. 2.**
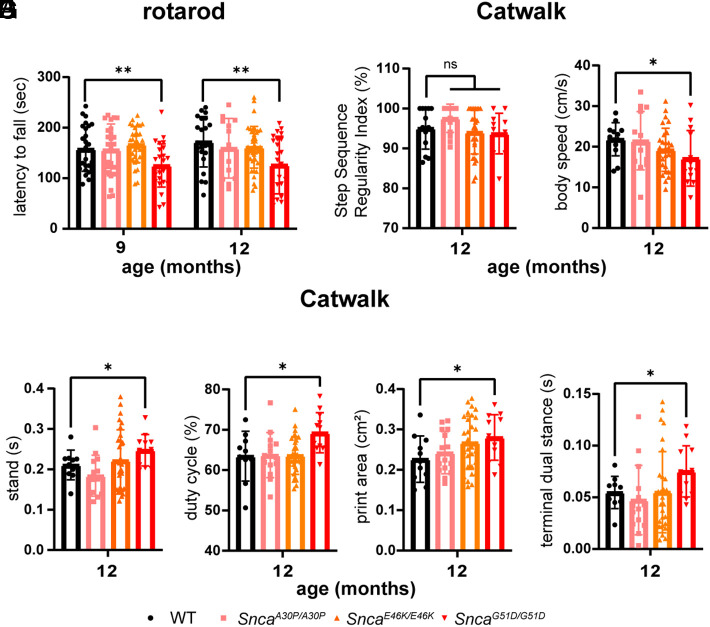
Characterization of the motor incoordination of knock-in mice harboring one of three different α-Syn mutations. (*A*) Latency to fall from the rotating rod. WT (n = 33: n = 17 male and n = 16 female), *Snca^A30P/A30P^* (n = 14: n = 7 male and n = 7 female), *Snca^E46K/E46K^* (n = 37: n = 18 male and n = 19 female), and *Snca^G51D/G51D^* (n = 29: n = 15 male and n = 14 female). (*B*–*G*) *Snca^G51D/G51D^* mice show altered gait on the catwalk assay, although step sequence which indicates inter-limb coordination is still normal (*B*), body speed (*C*), stand (*D*), duty cycle (*E*), average printed area (*F*), and terminal dual stance (*G*) of the hind limbs. WT (n = 10: n = 5 male and n = 5 female), *Snca^A30P/A30P^* (n = 14: n = 7 male and n = 7 female), *Snca^E46K/E46K^* (n = 30: n = 15 male and n = 15 female), and *Snca^G51D/G51D^* (n = 14: n = 7 male and n = 7 female). *P < 0.05, **P < 0.01, and n.s., not significant.

There were no significant differences in the pole test or grip strength test (Fig. 1 *D* and *E*), but the *Snca^G51D/G51D^* mice showed greater incoordination on the parallel rod floor test, with a greater number of footslips (Fig. 1*F*), and they also had a shorter latency to fall on the rotarod (Fig. 2*A*). In the catwalk assay and footprint analysis, performed at 12 mo of age, the *Snca^G51D/G51D^* mice did not show the altered index of gait coupling of sequence, which indicates interlimb coordination is normal (Fig. 2*B*), but their average movement speed was slower (Fig. 2*C*). The *Snca^G51D/G51D^* mice kept their paws on the ground longer (Fig. 2*D*), had a greater duty cycle (stance duration relative to step cycle duration) (Fig. 2*E*), total print area (Fig. 2*F*), and terminal dual stance (length of time both hind paws stayed on the ground) than WT mice (Fig. 2*G*). These findings indicate that the mutant mice, similar to PD patients, have greater difficulty lifting their feet and maintaining smooth motion and balance. As most of the key motor behavior phenotypes are not significantly altered in *Snca^A30P/A30P^*, *Snca^E46k/E46K^*, and *Snca^G51D/+^* mice (*SI Appendix*, Fig. S2 *A*–*F*), we focus our in-depth analyis on the *Snca^G51D/G51D^* mice for the rest of this paper.

### Homozygous *Snca*^*G51D*^ Mice Develop Pathological Species of α-Syn.

**Fig. 3.**
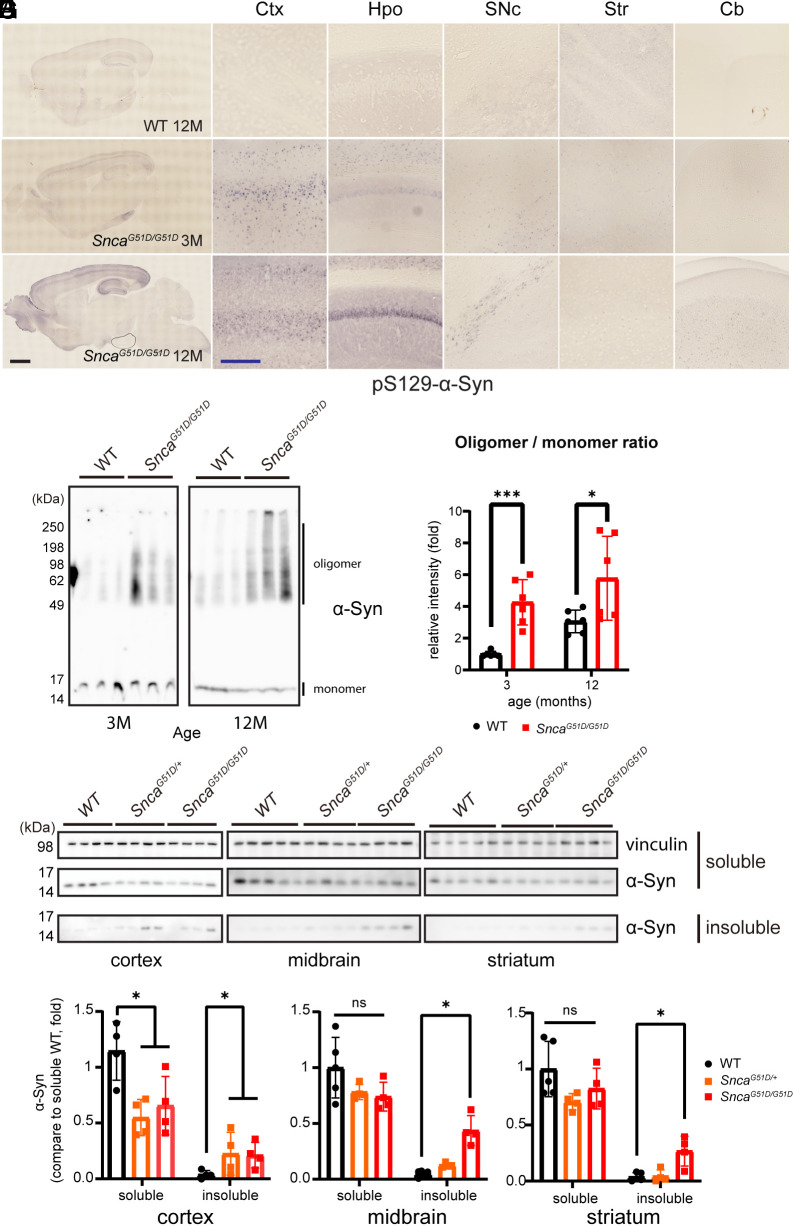
Abnormal α-Syn phosphorylation and formation of oligomers and detergent-insoluble α-Syn aggregates in homozygous *Snca^G51D^* mice. (*A*–*C*) Brain distribution of pS129-α-Syn in WT (*A*, 12 mo old) and *Snca^G51D/G51D^* mice (*B*, 3 mo old, and *C*, 12 mo old). Higher magnification of *A*–*C* in the cortex (ctx), hippocampus (hpo), striatum (str), substantia nigra pars compacta (SNc), and cerebellum (Cb). (Scale bar black: 1 µm and blue: 250 µm.) (*D*) Blue native page gel image of oligomeric α-Syn at 3 and 12 mo in WT and *Snca^G51D/G51D^* mice whole-brain lysates. (*E*) Quantification of oligomeric α-Syn protein intensities compared to monomeric α-Syn. n = 6, ***P < 0.005, *P < 0.05. (*F*) Immunoblot of 1% Triton X-100 soluble and insoluble α-Syn in the cortex (ctx), striatum (str), and midbrain (mid) from WT, *Snca^G51D/+^,* and *Snca^G51D/G51D^* mice. (*G*) Quantification of 1% Triton X-100 soluble and insoluble α-Syn intensities compared to WT soluble α-Syn. n = 4 to 5, *P < 0.05, ns, not significant.

Phosphorylation at Serine 129 (pS129-α-Syn) is a major pathological posttranslational modification of α-Syn seen in postmortem human PD tissue (30, 31). We examined *Snca^G51D/G51D^* mice at two ages (3 and 12 mo) to determine whether pS129-α-Syn was present and in which brain regions. At 3 mo, the cortex and hippocampus showed the strongest pS129-α-Syn signals (Fig. 3 *A*–*C*). At 12 mo, α-Syn phosphorylation was apparent in the cortex, hippocampus, and substantia nigra, but low in the striatum and cerebellum (Fig. 3 *A*–*C* and *SI Appendix*, Fig. S3*A*). In the cortical region of the 12-mo-old brain, pS129-α-Syn signals were detected in the anterior cingulate cortex, the motor cortex, and the somatosensory cortex (*SI Appendix*, Fig. S3*B*). In contrast to transgenic mice overexpressing α-Syn under Thy1 promoter [Thy1-α-Syn TG line 61 (20)], which exhibit pS129-α-Syn signal in all layers of the cortex, our *Snca^G51D/G51D^* mice displayed α-Syn phosphorylation in only two layers of the cortex (*SI Appendix*, Fig. S3*B*). We validated the layer-specific pS129-α-Syn signal using Ctip2 and found the signal in layers II and IV at 3 mo of age, expanding to layer V at 12 mo of age (*SI Appendix*, Fig. S3*C*).

Phosphorylated α-Syn makes toxic soluble oligomeric species (32, 33) as well as insoluble aggregates (34, 35). We therefore measured α-Syn oligomers in G51D mice by running the protein extracts on the blue native polyacrylamide gel electrophoresis (BN-PAGE) system. Both 3- and 12-mo-old mice showed soluble oligomeric species of α-Syn in BN-PAGE analysis at molecular weights from 60 kDa to higher than 250 kDa (Fig. 3 *D* and *E*). Next, we measured the detergent (1% Triton X-100)-insoluble aggregates of α-Syn protein by separating soluble/insoluble fractions from brain lysates using TritonX-100 or sodium dodecyl sulfate (SDS). In this assay, the mutant mice displayed TritonX-100-insoluble α-Syn aggregates in the cortex for both hetero- and homozygous *Snca^G51D^* mice. Additionally, significant increase of insoluble aggregates only in *Snca^G51D/G51D^* mice in the midbrain and striatum at 12 mo of age (Fig. 3 *F* and *G*). Therefore, *Snca^G51D/G51D^* (hereafter the G51D mice) thus form three pathological species of α-Syn: phosphorylated species, soluble oligomeric species, and aggregates.

### G51D Mice Show Early Phosphorylation of α-Syn in Olfactory Tissues along with Functional Deficits.

**Fig. 4.**
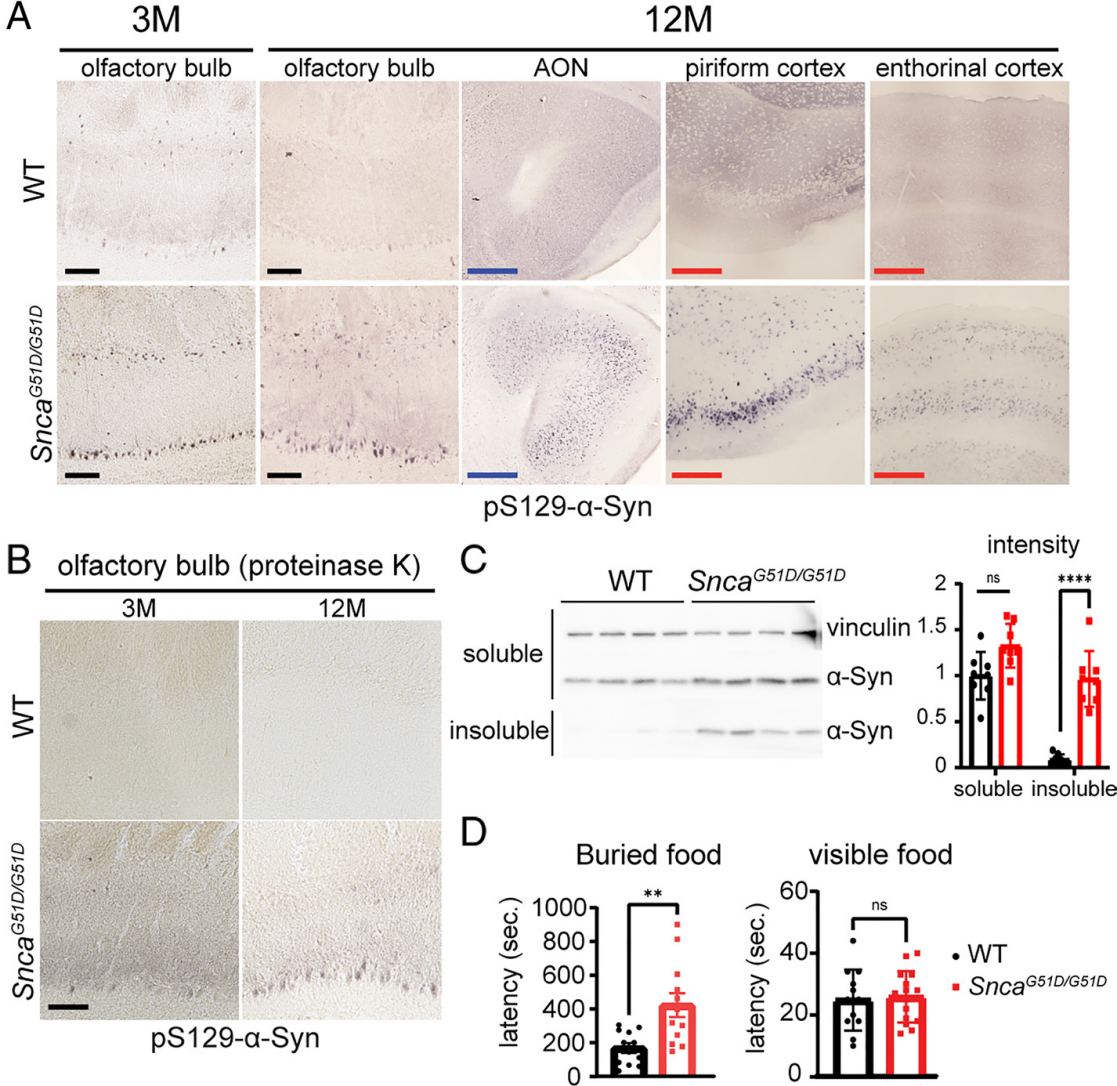
G51D mice show phosphorylated α-Syn along the olfactory tract and impaired olfaction. (*A*) Phosphorylated α-Syn in the olfactory bulb and different tissues along the olfactory circuit. of WT and *Snca^G51D/G51D^* (G51D) mice. [Scale bar (black): 100 μm, blue: 500 μm, and red: 200 μm.] (*B*) Proteinase K–resistant α-Syn is apparent in the olfactory bulb of 3- and 12-mo-old G51D, but not in WT control. (*C*) Detergent-insoluble α-Syn in the olfactory bulb of 12-mo-old mutant mice, but not in WT control by western blot (*Left*) and quantification of α-Syn protein intensities (*Right*). ****P < 0.0001 and ns, not significant. n = 8 for each genotype. (*D*) Olfaction assessment at 6 mo in WT (n = 12: n = 6 male and n = 6 female) and G51D mice (n = 12: n = 6 male and n = 6 female). Latency to find the buried (*Left*) or visible food (*Right*). **P < 0.01 and ns, not significant.

Braak staging of PD shows that the earliest Lewy body deposition is in the olfactory bulb, and nearly all (95%) of PD patients suffer impaired olfaction 5 y prior to the onset of motor symptoms (9, 36). To avoid the staining of activity-dependent phosphorylation of α-Syn (37, 38), we used more diluted pS129-α-Syn antibody (*Material and Methods*) to detect pathologic pS129-α-Syn. Our G51D mice showed pS129-α-Syn in the olfactory bulb in young adulthood (3 mo) as well as at 12 mo of age (Fig. 4*A*). We next examined brain regions transversed by the olfactory tract neurons to connect the olfactory bulb to the hippocampus (anterior olfactory neurons, piriform cortex, and entorhinal cortex). All these regions in G51D mice showed greater pS129-α-Syn staining than WT at 12 mo. At 12 mo, they also showed proteinase K-resistant (Fig. 4*B*) and Triton X-100 insoluble (Fig. 4*C*) α-Syn protein.

To see whether the mice showed concomitant deficits in olfaction, we performed a buried food and visible food pellet test on 6-mo-old WT and G51D mice. The mutant mice took significantly longer to find the buried food pellet than the WT mice (Fig. 4 *D*, *Left*) but did not differ from WT in obtaining visible food pellets (Fig. 4 *E*, *Right*). The mutant mice therefore did not have difficulty finding the buried pellet because of lack of appetite. The G51D mice show olfactory deficits similar to what occurs in human PD patients.

### Phosphorylation of α-Syn in the Enteric and Vagus Nerves of G51D Mice Correlates with Delayed Gut Transit.

**Fig. 5.**
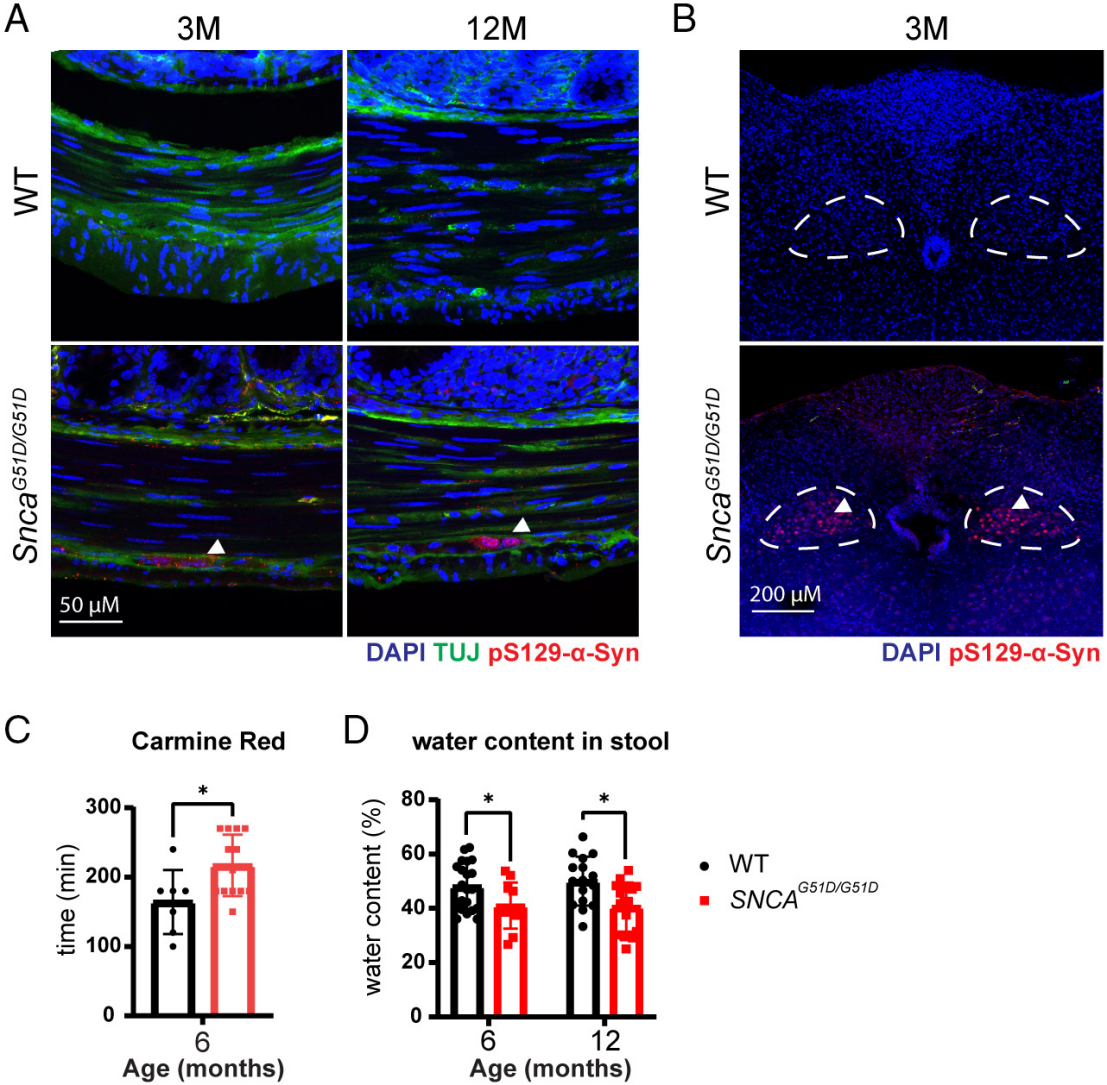
Accumulation of phosphorylated α-Syn in the ENS accompanies prolonged gut transit times in G51D mice. (*A*) Staining of pS129-α-Syn in the enteric nerve of WT and G51D mice. White arrow: pS129-α-Syn signal. (Scale bar: 50 µm.) (*B*) Representative image of the medulla oblongata of WT and mutant mice. White dash line: DMV area. White arrow: pS129-α-Syn-positive cells in DMV. White bar: 200 µm. (*C*) Carmine Red assay for total gut transit time measurement. WT (n = 7: n = 4 male and n = 3 female) and G51D mice (n = 13: n = 7 male and n = 6 female), *P < 0.05. (*D*) Water content in stool for 6- and 12-mo-old mice. n = 14 for each, *P < 0.05.

Approximately 20 y prior to the onset of clear motor symptoms, PD patients experience slow gut transit time and constipation (39, 40). Consistent with this clinical symptom and with Braak’s hypothesis, human postmortem studies indicate that the ENS is the first place to show phosphorylated α-Syn (41, 42) and that pathology spreads to the central nervous system through the vagus nerve (9). By the age of 3 mo, the G51D mice showed detectable levels of pS129-α-Syn in the enteric nerves of the colon (Fig. 5*A*) and the DMV (Fig. 5*B*), without a significantly reduced number of total DMV neuron numbers (*SI Appendix*, Fig. S4 *A*–*C*). To determine whether gut motility is diminished, we measured whole gut transit time by oral gavage of Carmine Red dye and found that the mutant mice took longer to produce dye-bearing stool (Fig. 5*C*). Fecal pellets from the mutant mice also contained less water than those from WT controls, suggestive of prolonged residence time in the colon (43) (Fig. 5*D*). These findings are consistent with symptoms observed in PD patients.

### G51D Mice Show Astrogliosis and Microgliosis But No Glial α-Syn Inclusions in the Cortex, Striatum, or Substantia Nigra.

**Fig. 6.**
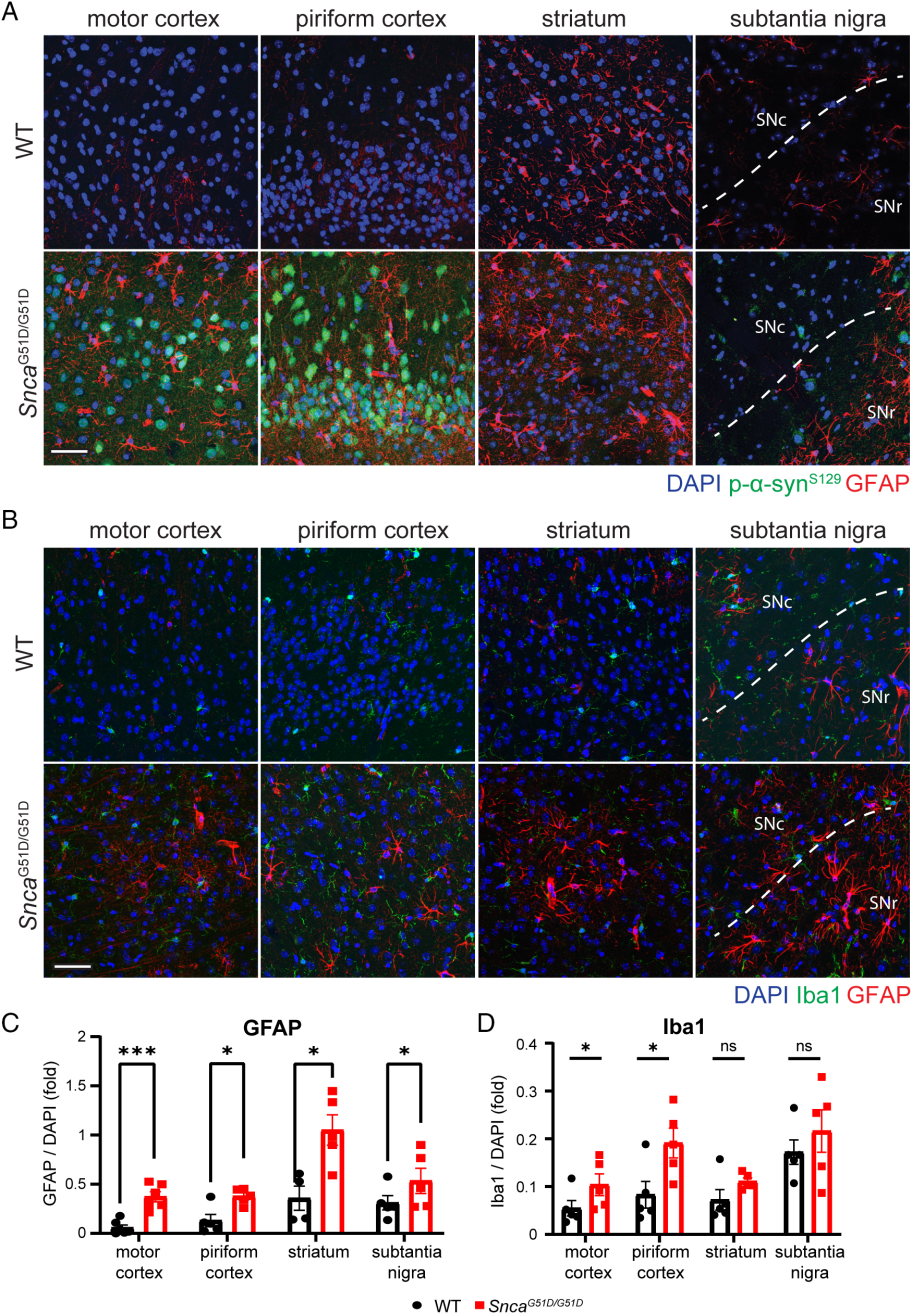
Astrogliosis and microgliosis in different brain regions of G51D mice. Glial activation is assessed in 12-mo-old WT (n = 4 to 6) and G51D mice (n = 4 to 6). [Scale bar (white): 50 µm.] (*A*) Representative images of astrocyte activation in the motor cortex, piriform cortex, striatum, and substantia nigra visualized with pSer129-α-Syn and GFAP double immunostaining. (*B*) Representative images of microglia activation in the motor cortex, piriform cortex, striatum, and substantia nigra visualized with pSer129-α-Syn and Iba1 double immunostaining. (*C* and *D*) Relative quantities of GFAP (*C*) and Iba1 (*D*) signal intensities compared to DAPI. WT n = 4~6 for WT and G51D mice. *P < 0.05, ***P < 0.005, and ns, not significant.

Astrogliosis and microglia activation are common inflammation-related neuropathologies observed in PD and many other neurodegenerative diseases (44–48). Our G51D mice showed astrogliosis by Glial fibrillary acidic protein (GFAP) immunopositivity in the motor cortex, piriform cortex, striatum, and reticular region of the substantia nigra at 12 mo (Fig. 6 *A* and *C*). Microglial activation, indicated by increased Iba1 staining, was also apparent in the motor cortex and piriform cortex (Fig. 6 *B* and *D*).

Previous studies have reported that PD patients carrying the *SNCA^G51D^* mutation display pathology similar to MSA (15, 19), with oligodendrocyte α-Syn inclusions, loss of oligodendrocytes, and astrocyte/microglia activation (49). We assessed the neuron and glia markers with pS129-α-Syn labeling in G51D mice and their littermate controls. Most of the pS129-α-Syn colocalized with neuron markers (NeuN), whereas no astrocytes (GFAP) and few (<5%) oligodendrocytes (Olig2) showed a pS129-α-Syn signal (*SI Appendix*, Fig. S5 *A*–*D*). This suggests that α-Syn pathology in G51D mice resembles PD more than MSA.

### G51D Mice Show Loss of Dopaminergic Neurons in the Substantia Nigra and Noradrenergic Neurons in Locus Coeruleus (LC).

**Fig. 7.**
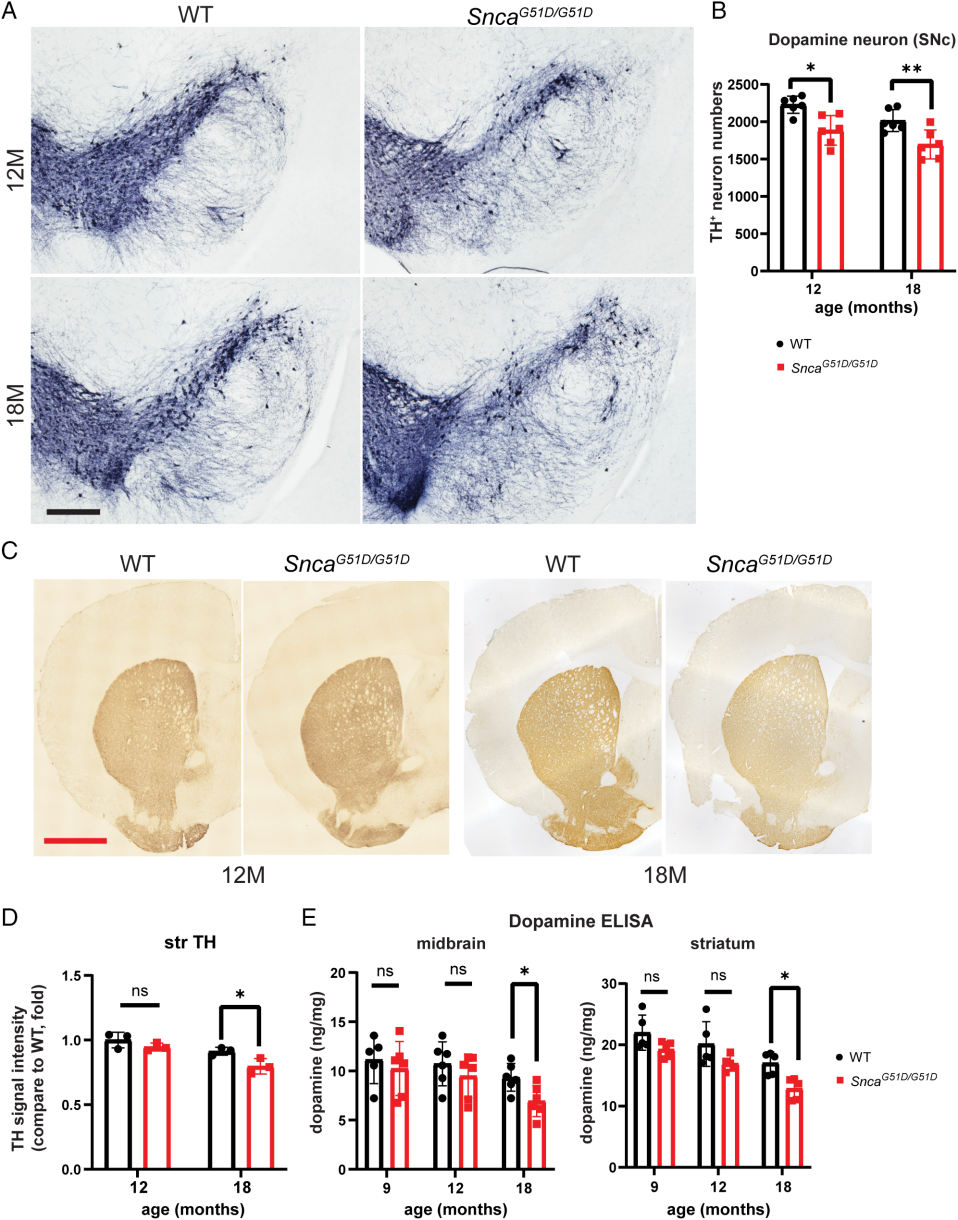
Loss of dopaminergic neurons in G51D mice. (*A*) Representative image of TH-positive dopaminergic neurons in the substantia nigra of 12- and 18-mo-old WT control and G51D mice. [Scale bar (black): 400 µm.] (*B*) Quantification of TH-positive cells in the substantia nigra of 12- and 18-mo-old WT control and G51D mice. n = 6 for each. (*C*) Representative image of TH-positive dopaminergic neurites in the striatum of 12- and 18-mo-old WT control and G51D mice. [Scale bar (red): 1,000 µm.] (*D*) Quantification of TH-positive intensities in the striatum region of 12- and 18-mo-old WT control and mutant mice. n = 3 for each. (*E*) ELISA assessment of dopamine concentration in the midbrain (*Left*) and striatum (*Right*) regions of 9-, 12-, and 18-mo-old WT and mutant mice. n = 6 (midbrain) or 4 (striatum) for each, *P < 0.05, **P < 0.01, and ns, not significant.

Both PD patients and mouse models that overexpress α-Syn or injected with α-Syn PFF show loss of dopaminergic neurons in the substantia nigra (50, 51). We assessed dopaminergic neurons in our mutant mice by measuring tyrosine hydroxylase (TH) staining. G51D mice showed behavioral deficits at 12 mo of age, at which time there was mild loss of TH-positive staining in the substantia nigra that worsened with further aging (Fig. 7 *A* and *B*). We used tyrosine hydroxylase TH staining to assess dopaminergic neurites in the striatum of 12- and 18-mo-old G51D KI mice. Compared to WT controls, we observed no significant difference in TH staining intensity at 12 mo and a slight decrease at 18 mo in homozygous G51D KI mice (Fig. 7 *C* and *D*). Dopamine levels in the midbrain and striatum also decreased at 18 mo (Fig. 7*E*). We additionally evaluated the number of TH-positive neurons within the LC to detect whether noradrenergic neurons are decreasing, given the loss of such neurons as seen in PD patients (9, 52, 53). At 12 mo, G51D mice showed mild loss of noradrenergic neurons in LC regions (*SI Appendix*, Fig. S6 *A*–*C*). These results indicate that G51D mice show dopaminergic neuronal loss, reduction of dopamine production, and noradrenergic neuronal loss, all seen in PD patients.

## Discussion

Most genetically engineered mouse models for α-Syn-driven PD use neuronal promoters such as prion or Thy1. These promoters overexpress α-Syn in the substantia nigra, leading to dopaminergic neuron dysfunction with PD-like motor and nonmotor behavioral deficits (54–56). However, the limitation of these models is that this broad overexpression pattern leads to pathology throughout the brain, as the mice do not reflect endogenous α-Syn levels and tissue distribution. This G51D mouse model enabled us to dissect the time-dependent cellular vulnerability and the features that accompany the early stages of the disease before the development of motor symptoms.

In our G51D mice, the initial deposition of α-Syn phosphorylation in the olfactory tract, vagal, and enteric nerves—similar to Braak stages I and II—takes place from 3 to 6 mo of age. Then α-Syn pathology spreads along the olfactory circuit to the piriform cortex, entorhinal cortex, and hippocampus CA2, and from the vagus nerve connects to the substantia nigra in the midbrain (57)— similar to Braak stages III and IV—with motor incoordination appearing at 9 to 12 mo (*SI Appendix*, Fig. S7). Another neuropathology in PD postmortem tissue from patients harboring either duplication of the *SNCA* region, *SNCA^A53E,^* or *SNCA^G51D^* mutation, is the Lewy body deposition in layers II and IV in the cortical tissues, which are the same layers showing pathology in our mice (58, 59). The G51D mice also showed pS129-α-Syn in the motor cortex, whose Layers II and V harbor a dense population of pyramidal neurons that connect the cortex to various subcortical structures necessary for motor coordination, including the striatum, pons, brainstem, and spinal cord (60). These mice therefore replicate the pattern of pathology and progression typical of human PD quite well.

Still, future studies are needed to understand what drives the delayed increase in α-Syn phosphorylation, which is not detectable at 3 mo (outside of the olfactory system) and becomes apparent after 12 mo of age in the deeper layer of the cortex, hippocampus, and substantia nigra in the G51D mice. Previous studies suggested that the high expression of *SNCA* in the hippocampus and relatively strong expression in the substantia nigra drive such accumulations (61–63). Dissecting this further is key to determining whether there is a component of tissue-specific abundance underlying α-Syn accumulation that could be independent of potential tissue-to-tissue transmission.

Another question pertains to the relationship between motor deficits and dopamine loss. Our mice display motor deficits from 9 mo but do not show a significant reduction in dopamine levels until 18 mo of age. Similarly, *Snca* transgenic mouse models show motor incoordination starting from 2 to 9 mo of age but do not show loss of dopamine until 14 to 18 mo (20, 21, 64–66). These findings suggest that dopaminergic neuronal dysfunction rather than neuronal loss is the early driver of motor deficits. Cellular stress, such as neuroinflammation, seems to coincide better with dopaminergic neuron dysfunction. Previous studies support this notion, as behavioral changes in α-Syn transgenic mice are often accompanied by increased reactive astrocytes (67–69) or activated microglia (70). These data suggest that neuroinflammation and neuronal dysfunction disrupt motor function in PD mice models well before significant dopamine loss.

Although we did not conduct further studies on A30P and E46K KI mice, these mice may exhibit molecular or behavioral phenotypes at a later stage. A30P KI mice showed some differences in anxiety-related behavior at 9 mo and grip strength tests at 12 mo compared to all combined WT controls, but these differences were not significant when comparing A30P KI mice to their own WT littermate mice only (data not shown). However, we observed an increase in phosphorylated α-Synuclein in both A30P and E46K KI mice. Although this increase was lower than in G51D mice, it was still significantly different from WT mice, suggesting that A30P and E46K KI mice could develop motor deficits after 12 mo of age.

There are only a few studies of G51D in animal models with limited analyses, so it is early to compare our G51D KI mouse with G51D KI rat or G51D transgenic mouse lines. A positron emission tomography study of the rat G51D KI model found increased, asymmetrical dopamine turnover in the striatum of aged animals, which is also found in humans, but the study did not evaluate olfactory function, gut motility, behavioral phenotypes, or phospho-S129-α-Syn pathology (66). A G51D transgenic mouse model shows motor deficit at 6 mo and degeneration of the substantia nigra at 12 mo, again without reporting on any of the nonmotor phenotypes (24). One possibility is that the transgenic mice might not recapitulate all the phenotypes we detected due to the use of a heterologous promoter that might not recapitulate the endogenous regional abundance of *Snca*.

In summary, the G51D KI mouse model expresses mutant α-Syn^G51D^ in the native spatiotemporal pattern and displays the cascade of molecular events and symptom progression characteristic of human PD, most notably with olfactory and enteric dysfunction preceding motor dysfunction by months. As a genetic PD mouse model that accurately recapitulates nonmotor PD symptoms, this model will be useful for investigating molecular pathogenesis underlying PD and testing therapeutic options to reduce toxic α-Syn.

## Materials and Methods

### Animals.

All mice were housed in a level 3, American Association for Laboratory Animal Science–certified facility on a 14-h light cycle. Husbandry, housing, killing of animal, and experimental guidelines were approved by the Institutional Animal Care & Use Committee at Baylor College of Medicine.

### Generation of α-Synuclein Familial Mutant Knock-In Mice.

*Snca^A30P^*, *Snca^E46K^*, and *Snca^G51D^* knock-in mice were generated via CRISPR/CAS9-mediated standard homologous recombination methods in the Genetically Engineered Rodent Models Core at Baylor College of Medicine, as previously described (71). In brief, we designed crRNA 20 nucleotides upstream of the protospacer-adjacent motif (PAM) site to a region of interest using crispr.mit.edu. To design ssODN for point-mutation knock-in, we insert a point mutation flanked by 6 to 8 synonymous mutations (ideally AT to GC to get a high Tm for genotyping), followed by flanking with a 75-nucleotide left homology arm and a 100-nucleotide right homology arm. This is designed to destroy the PAM or a guide RNA (gRNA) sequence with synonymous mutations to prevent recutting and to allow for genotyping by differential primer hybridization. See the details in *SI Appendix*.

### Behavioral Assays.

We tested 6-, 9-, and 12-mo-old homozygous *Snca^A30P^*, *Snca^E46K^*, and *Snca^G51D^* mice with its wild-type littermates. Mice were acclimated to the test environments for 30 min before testing, with at least a 30-min interval between each trial. An equal number of male and female mice were used in this study.

#### Open-field test.

The open-field arena is a 40 × 40 × 40 cm (width × length × height) cubical enclosure. The central 20 × 20 cm region of the box was marked by ANY-maze software (Stoelting Co.). The mouse was placed in a corner of the box and recorded by an overhead video camera for 10 min. Several measures including total distance traveled, mean motor speed, and center/total ratio were analyzed.

#### Pole test.

The mice were placed head up near the top of a vertical pole (Sigma-Aldrich Z509442, pole substitute with fully threaded rod, 50 cm long, 1 cm in diameter). We counted the number of times they turned their head and whole body downward (turn) and climbed down to the ground (down) the pole during a maximal time of 60 s. The mice undertook three trials. The minimum durations of “turn” and “down” time measured and down time were used for analysis.

#### Grip strength.

A mouse was picked up by the base of a tail and gently lowered toward the net until it grasped the bar of the grip strength meter. (Columbus Instruments 0167-8001). The mouse was then gently pulled backward until it released its grip. The maximum pull force at the time the animal released the grip was recorded on a horizontally mounted scale equipped with a drag pointer. Mice underwent three trials. The maximum score across the three trials was used for the analysis.

#### Parallel rod floor test.

Animals were placed individually into the center of a wire grid laid within an open-field chamber (Accuscan) for 10 min. The number of paw slips through the wire grid was recorded and analyzed using ANY-maze (Stoelting). The number of foot slips was normalized to the total distance traveled.

#### Rotarod.

Mice were placed on an accelerating rotarod apparatus (Type 7650, Ugo Basile) and allowed to move freely as the cylinder increased from 5 rpm to 40 rpm over a 5-min period. Each trial lasted a maximum of 10 min, and the latency to fall was measured when the mouse fell off the rod or rode the cylinder for two consecutive revolutions without regaining control. The maximum cutoff time of the test was 600 s. Each training day consisted of four attempts with a 30 min rest in between each trial for a period of 3 d, and data were used for day 3.

#### Gait analysis.

All gait analysis recordings and paw print identification were done using the CatWalk XT hardware and software (Noldus). The apparatus consists of a 130-cm tunnel, with a glass floor with an internally reflected green light and a ceiling that contains a red light to provide contrast to the internally reflected green light. The pattern of green light is interpreted by custom software and translated into a quantitative assessment of rodent paw movements. Each mouse was given a trial (or run) that was considered successful if the duration was between 0.8 and 5 s and performed at a steady speed (<60% variation). Mice were given a maximum of 10 consecutive attempts to make three successful crossings. See the details in *SI Appendix*.

#### Buried food pellet hunt.

In a box, we made one-inch-thick bedding and placed the mouse in the box for 15 min a day for 3 d to habituate them. At the end of the third day, we weighed the mouse and restricted its food. Exactly 24 h after food restriction, we buried a regular food pellet 3 mm below the bedding surface, then placed the mouse in the center of the box and measured the time it takes for it to find and eat the food. We then placed the mouse in a box with a visible food pellet the next day and measured the time it took them to eat it, as an indicator of their basal food-seeking ability.

#### Whole gut transit time.

We administered 6% carmine red solution by oral gavage (150 μL, in a 1 mL syringe attached to a feeding needle) to the mice and recorded the time it took to pass in fecal pellets. We transferred each mouse individually into a sterile cage, checked every 10 min that the mice had a fresh fecal pellet, and measured the time it took for each mouse to release dye-stained pellets.

#### Water content in the stool.

Each mouse was placed in a separate clean cage and observed throughout the 60-min collection period. Fecal pellets were collected immediately after expulsion and placed in sealed 1.5 mL tubes to avoid evaporation. Tubes were weighed to obtain the wet weight of the stool, which was then dried overnight at 55 °C and reweighed to obtain the dry weight. The stool water content was calculated from the difference between the wet and dry stool weights.

### Protein Extraction and Western Blot.

Mice were killed by isoflurane inhalation at 3, 12, and 18 mo of age. The olfactory bulb, cortex, and midbrain region containing the substantia nigra were harvested and immediately frozen on dry ice. Samples were mixed with 5 to 10 volumes of modified radioimmunoprecipitation assay (RIPA) buffer (50 mM Tris-Cl, 150 mM NaCl, 1% NP-40, 0.5% sodium deoxycholate, and 0.1% SDS) supplemented with 0.5% triton X-100, 1× protease inhibitor, and 1× phosphatase inhibitor buffer and lysed with a sonication step (20 pulses, output 2.5, duty cycle 30%, 2 s and 2 s rest intervals, fives times). Samples were centrifuged at 20,000 g for 20 min, and the supernatant was collected for use.

### SDS-PAGE and Western Blot.

Protein samples were loaded on either 10- or 15-well NuPAGE 4 to 12% Bis-Tris gels (Thermo Fisher Scientific). Gels were run in 1× 2-(N-morpholino)ethanesulfonic acid (MES)/SDS protein running buffer and transferred onto nitrocellulose membranes in Tris-Glycine buffer (25 mM Tris and 190 mM glycine) supplemented with 10% methanol at 0.3 A for 1.5 h. After being transferred, membranes were blocked in 5% milk in Tris-buffered saline with 0.1% Tween 20 detergent (TBS-T) for 1 h and probed with one of the following primary antibodies overnight: mouse anti-vinculin (1:10,000), mouse anti-α-Syn (1:3,000), and rabbit anti-pS129-α-Syn (1:3,000). Membranes washed three times in TBS-T for 10 min and add secondary mouse or rabbit HRP-conjugated secondary antibodies were applied in 5% skim milk in TBST. Following the wash, chemiluminescence was induced by ECL (GE Healthcare, RPN2236) and imaged by Amersham imager 680 (GE Healthcare).

### Triton X-100 Soluble/Insoluble Fractionation.

Twelve-month-old wild-type and *Snca^G51D/G51D^* mouse cortices, midbrain, striatum, and olfactory bulbs were lysed by applying 400 μL of Phosphate-buffered saline (PBS) with 1% triton X-100. Lysates were transferred into a Beckman Coulter VWR tube (BK357448), followed by ultracentrifugation at 110,000 g with TLA-110 rotor for 60 min at 4 °C. Triton X-100–soluble supernatant was carefully transferred. Triton X-100–insoluble pellets were washed with 1 mL of PBS and removing all the supernatant. A volume of 100 μL of 2% SDS was added to pellets and dispersed by pipetting, probe-sonicated using a sonicator (Branson Sonifier 450), and ultracentrifuged at 110,000 g with a TLA-110 rotor for 60 min at 4 °C. Triton X-100–soluble and –insoluble (2% SDS soluble) samples were western blotted as described above. To match the relative amount of protein, we expose the blot four times longer for the insoluble fraction compared to the soluble fraction.

### Native Gel Western Blot.

For Native-PAGE experiments, 3- and 12-mo-old wild-type and *Snca^G51D/G51D^* mice cortices were lysed following the instruction of the NativePage Sample kit (Thermo Fisher Scientific, BN2008). Samples are homogenized with 1× NativePage sample buffer supplemented with 1% n-Dodecyl-Beta-Maltoside (DDM) and centrifuged in 20,000 g for 20 min, and supernatants were collected and loaded without boiling and directly onto NativePage 4 to 16% Bis-Tris protein gels (Thermo Fisher Scientific, BN1004). After transfer to the membrane, samples were done with western blot performed as described above.

### Immunofluorescence (IF) and Immunohistochemistry (IHC).

Tissue preparation and immunostaining were done as previously described (72). For IF and immunohistochemical experiments, mice were transcranially perfused with PBS followed by 4% paraformaldehyde (PFA). Brains were dissected and fixed in 4% PFA for 2 d and dehydrated for 24 h in 15% sucrose (w/v, in PBS) followed by a 2-d incubation in 30% sucrose solution (in PBS), all at 4 °C. The brains were then frozen on dry ice in Optimal cut temperature (OCT) compound (VWR, 25608-930) and sectioned on a cryostat (Leica CM 3050S). Sections were collected at 40 µm thickness. Sections were kept in 1× PBS with 0.01% NaN_3_ until use. For the antibody titer and details, see the Immunofluorescence (IF) and immunohistochemistry (IHC) section in *SI Appendix*.

### Proteinase K Treatment of the Brain.

The sections for immunostaining were incubated with a solution of 0.05% SDS and 10 µg/mL Proteinase K (Thermo Fisher Scientific, EO0491) for 5 minutes at 37°C. After incubation, the sections were blocked with 5% normal goat serum (NGS) for 1 hour at room temperature, followed by the standard IHC process (73).

### Counting of Dopaminergic and Noradrenergic Neurons.

Stereological counts were performed as previously described (72). Briefly, sections from +0.26 to −0.4 (for dopaminergic neurites in str), −2.54 to −4 (for dopaminergic neurons in SNc), and −4.8 to −5.8 from Bregma (for Adrenergic neurons in LC) were selected for staining in intervals of six. IHC for TH was performed as described above. All IHC for stereology was imaged using 40× (the representative images were taken using 10× objective) on a Ti2E Spinning Disc confocal microscope (Nikon). The experimenter outlined the SNc or Str and counted TH-positive cell bodies (SNc, LC) or intensities (Str). The total number of cells was estimated using the measured tissue thickness.

### Dopamine Level Quantification in the Midbrain Using the ELISA.

Midbrain tissue containing substantia nigra regions and striatum tissues are obtained from 9-, 12-, and 18-mo-old WT and *Snca^G51D/G51D^* mice, and dopamine levels in the tissues are determined by using a commercial ELISA kit for dopamine (Enzo Lifescience) according to the manufacturer’s instructions.

### Statistical Analyses.

For all experiments, comparisons between two groups were conducted using Student’s t-test. When the group sizes were significantly different, an unequal variance t-test was used (Figs. 1 and 2 *C*–*F*). For comparisons involving three or more groups, one-way ANOVA followed by Dunnett’s multiple comparison test was performed. All analyses were conducted using Prism 10 software (GraphPad).

## Supplementary Material

Appendix 01 (PDF)

## Data Availability

All study data are included in the article and/or *SI Appendix*.
