# Supplementary material for: Olfactory deficit and gastrointestinal dysfunction precede motor abnormalities in alpha-Synuclein G51D knock-in mice
Source: Proc Natl Acad Sci U S A. 2024 Sep 16;121(39):e2406479121. doi: 10.1073/pnas.2406479121 (PMC11441490; doi:10.1073/pnas.2406479121)
Supplement: Supplementary file 1 — Appendix 01 (PDF) [file pnas.2406479121.sapp.pdf]

Supplemntal Material and Methods

Generation of α-Synuclein familial mutant knock-in mice

Mice were bred in C57BL/6J background. We designed crRNA 20 nucleotides upstream of the PAM site to a region of interest using crispr.mit.edu. To design ssODN for point-mutation knock-in, we insert a point mutation flanked by 6-8 synonymous mutations (ideally AT to GC to get a high Tm for genotyping), followed by flanking with a 75-nucleotide left homology arm and a 100-neucleotide right homology arm. This is designed to destroy the PAM or gRNA sequence with synonymous mutations to prevent re-cutting and to allow for genotyping by differential primer hybridization. We mixed 40ng of crRNA with 40ng Alt-R® CRISPR-Cas9 tracrRNA, 1µg of ssODN and 30ng of Alt-R® S.p. HiFi Cas9 Nuclease V3 in 100µl of $T_{10}E_{0.1}$ buffer before injection into mouse embryos. All the reagents were ordered from Integrated DNA Technologies, Inc. (USA). Knock-in mice were confirmed by genomic PCR with different primers between the unmodified (WT) and modified (A30P, E46K, and G51D) alleles (for the sequences, see the supplement Table 1).

Gait analysis

A detailed parameter analysis was following previous literature (1).

Gait parameters: The gait parameters examined with the CatWalk software in this study are described below (LF, left forepaw; LH, left hind paw; RF, right forepaw; RH, right hind paw).

Step pattern and Regularity index: A mouse can use six possible step sequence patterns as it places its four paws one after another. These patterns can be categorized into 3 groups: alternate (Aa: RF-RH-LF-LH; Ab: LF-RH-RF-LH; cruciate (Ca: RF-LF-RH-LH; Cb: LF-RF-LH-RH); and rotary (Ra: RF-LF-LH-RH; Rb: LF-RF-RH-LH). If the mouse step pattern is out of these six step sequences, it is considered abnormal. The regularity index expresses the ratio of normal step sequence patterns relative to the total number of paw placements. It represents inter-limb coordination during the gait cycle.

Body speed: This parameter is calculated by dividing the distance that the animal's body traveled from one initial contact of one paw to the next by the time to travel that distance

Stand: This parameter is a duration of contact of paw with glass

Duty cycle: This parameter is an indication of ratio of paws on the ground during one step cycle.

Print area: This parameter is a measure of the surface area of maximal contact of the paw with the ground (cm^2).

Terminal dual stance: This parameter is the duration of ground contact for both hind paws simultaneously, but the latter one in a step cycle of a target hind paw.

Immunofluorescence (IF) and immunohistochemistry (IHC)

Immunofluorescence: Immunofluorescence was performed as previously described (2). Briefly, floating sections were washed 3 times in 1X PBS, and then incubated with one or two of the following antibodies: rabbit anti-pSerS129-α-Syn antibody (1:1000), rat anti-Ctip2 antibody (1:200), goat anti-ChAT antibody (1:1000), mouse anti-NeuN Antibody (1:1000), rabbit anti-Iba1 (1:1000), goat anti-GFAP (1:1000), mouse anti-Olig2 (1:100), mouse anti-tubulin III (1:1000). Afterwards, the sections were washed 3 more times and stained with secondary antibodies with a designated fluorophore (488 or 555 nm) with 1:1000 concentrations overnight. After 3 times washing, 10min incubate with DAPI (Thermo Fisher Scientific, D1306, 1:200,000) for 10 min, and mount with Vectashield Hardset antifade mounting media (Vector Laboratories, H-1400-10). All imaging was performed on a confocal microscope (Leica STED TCS SP8X), using LAS X software (Leica) to select optimal settings for image capture. All IF images presented in this study were taken with the 10X and 40X objectives. All quantification was performed using Fiji software (3). Briefly, Z-stacks were loaded onto the software, which quantified the mean fluorescent signal and normalized to the DAPI signal before comparison.

Immunohistochemistry: Floating sections were washed 3 times in 1 X PBS and then incubated with PBS supplemented with 5% FBS, 0.3 % Triton X-100 one hour and incubated with antibodies as below overnight at 4ºC: rabbit anti-pSerS129-α-Syn antibody (1:10000 for olfactory bulb, 1:1000 for other tissue), rabbit anti-Tyrosine Hydroxylase antibody (1:1000). Afterwards, the sections were washed 3 more times and stained with the VECTASTAIN® Elite® ABC HRP Kit (Peroxidase, Rabbit IgG) (Vector laboratories, PK-6101) according to manufacturer's instructions. We then used the DAB Peroxidase (HRP) Substrate Kit (with Nickel), 3,3'-diaminobenzidine (Vector laboratories, SK-4100) to develop the

sections. Sections were mounted on Superfrost plus slides (Thermo Fisher Scientific, 22-037-246) and dried at room temperature. Slides were then dehydrated by incubating them in the following series of solutions: PBS, H2O, 70% Ethanol, 95% Ethanol, 100% Ethanol, and Xylene, before mounting coverslips using Richard-Allan Scientific™ Cytoseal™ XYL (Thermo Fisher Scientific, 8312-4).

Supplement table 1. Reagent and resources used for this paper

Reagent or resource	source	identifier
**Antibodies**		
Anti-Alpha-Synuclein antibody (clone 42)	BD	RRID: AB_398107
p-S129 Synuclein (clone EP1536Y)	Abcam	RRID: AB_869973
p-S129 Synuclein (clone D1R1R)	Cell Signaling Technology	RRID: AB_279886
Anti-Vinculin	Sigma-Aldrich	RRID: AB_477629
Anti-choline Acetyltransferase (ChAT, AB144P)	EMD Millipore	RRID: AB_2079751
Anti-Ctip2 antibody (clone 25B6)	Abcam	RRID: AB_2064130
Anti-NeuN Antibody (clone A60)	EMD Millipore	RRID: AB_2298772
Anti-GFAP	Novus Biologicals	RRID: AB_829022
Anti-Iba1	Wako Chemicals	RRID: AB_839504
Anti-Olig2 Antibody	EMD Millipore	RRID: AB_10807410
Anti-beta III-tubulin (TUJ, clone 2G10)	Sigma-Aldrich	RRID: AB_1841228
Anti-Tyrosine Hydroxylase (TH)	EMD Millipore	RRID: AB_390204
Alexa Fluor® 488 AffiniPure Donkey Anti-Mouse IgG (H+L)	Jackson ImmunoResearch	RRID: AB_2340846
Donkey anti-Rat IgG (H+L) Highly Cross-Adsorbed Secondary Antibody, Alexa Fluor™ 488	Thermo Fisher Scientific	RRID: AB_2535794
Donkey anti-Rabbit IgG (H+L) Highly Cross-Adsorbed Secondary Antibody, Alexa Fluor™ 488	Thermo Fisher Scientific	RRID: AB_2535792
Donkey anti-Rabbit IgG (H+L) Highly Cross-Adsorbed Secondary Antibody, Alexa Fluor™ 555	Thermo Fisher Scientific	RRID: AB_162543
Donkey anti-Goat IgG (H+L) Cross-Adsorbed	Thermo Fisher	RRID:

Secondary Antibody, Alexa Fluor™ 555	Scientific	AB_2535853
**Kit**		
Dopamine ELISA Kit	Enzo Lifescience	ENZ-KIT188-0001
VECTASTAIN(R) ELITE(R) ABC anti-Rabbit IgG HRP Immunodetection Kit	Vector Laboratories	Catalog #PK-6101
NativePage Sample kit	Thermo Fisher Scientific	Catalog #BN2008
**Experimental models: Organisms / strain**		
Mouse: wild type (WT: C57BL/6J)	Jackson Laboratory	Catalog #000664
Mouse: *Snca*^{A30P} KI	This paper	N/A
Mouse: *Snca*^{E46K} KI	This paper	N/A
Mouse: *Snca*^{G51D} KI	This paper	N/A
Mouse: B6;DBA-Tg(Thy1-SNCA)61Ema	Gift from Marie-Francoise Chesselet (UCLA)	MGI:5435401
**CRISPR-Cas9 reagents**		
Alt-R® CRISPR-Cas9 tracrRNA	IDT	catalog #1072532
Alt-R® S.p. HiFi Cas9 Nuclease V3	IDT	catalog # 1081060
**Oligonucleotides**		
crRNA for *Snca*^{A30P}:<br>5'- GGCAGCTGGAAAGACAAAAG-3'	IDT	N/A
crRNA for *Snca*^{E46K} / *Snca*^{G51D}:<br>5'- TTCCAAAACTAAGGAAGGAG-3'	IDT	N/A
ssODN for *Snca*^{A30P}:<br>5'-<br>GTTCATGAAAGGACTTTCAAAGGCCAAGGAGG<br>GAGTTGTGGCTGCTGCTGAGAAAACCAAGCAG<br>GGTGTGGCAGAaGCgcCgGGgAAaACgAAgGAGG<br>GAGTCCTCTATGTAGGTAGGTAGTGACACTGTG<br>ACTAATGAATTGGGGTGGCTGGTGTGTGGTGTC<br>TGATTCGTGTGCATCACAGCTTCTCAGAAGA-3'	IDT	N/A
ssODN for *Snca*^{E46K}:<br>5'-<br>GTACATTCCCTTCTGAATATTGAGAATTTTTAAT<br>TGGCTGCTGTAAATTGAAGGACAGTTTAATATT<br>TATGCGTTCAATTTCTTTGTTCTTTAGGTTCCAA<br>gACgAAaaAgGGcGTaGTgCAcGGgGTaACgACAGG<br>TAAGCTCTGTTGTCTTTTATCCAGGGGTGATATG<br>CCGAATGCCTTCTAGGCTAAATTAACTTG-3'	IDT	N/A
ssODN for *Snca*^{G51D}:<br>5'-<br>TGAGAATTTTTAATTGGCTGCTGTAAATTGAAG<br>GACAGTTTAATATTTATGCGTTCAATTTCTTTGT<br>TCTTTAGGTTCaAAgACgAAaGAgGGcGTGGTgCA	IDT	N/A

TGacGTGACAACAGGTAAGCTCTGTTGTCTTTTATCCAGGGGTGATATGCCGAATGCCTTCTAGGCTAAATTAACTTGATGCTTATACTTCAA-3’		
Genotyping primer for *Snca*^{A30P} and WT forward: anneal at 61 degree<br>5’-CACCCAAGAAATCACACTTACACCAGGACTTGG-3’	Sigma-Aldrich	N/A
Genotyping primer for *Snca*^{A30P} reverse: anneal at 61 degree<br>5’- CTTCGTTTTCCCCGGCGCTTCTG-3’	Sigma-Aldrich	N/A
Genotyping primer for WT sequence of *Snca*^{A30P} KI reverse: anneal at 61 degree<br>5’- CTTTTGTCTTTCCAGCTGCCTCT-3’	Sigma-Aldrich	N/A
Genotyping primer for *Snca*^{E46K} forward: anneal at 53 degree<br>5’- GGTGGAACAGCACAGGAG-3’	Sigma-Aldrich	N/A
Genotyping primer for WT sequence of *Snca*^{E46K} KI forward:<br>5’-AACTAAGGAAGGAGTGGTTCATGGAGTGACA-3’	Sigma-Aldrich	N/A
Genotyping primer for *Snca*^{E46K} reverse: anneal at 53 degree<br>5’- GGTCCATTGGTGATACCTGTAC-3’	Sigma-Aldrich	N/A
Genotyping primer for *Snca*^{G51D} forward: anneal at 63 degree<br>5’-AGGTGGAACAGCACAGGAGCTTCATACTGC-3’	Sigma-Aldrich	N/A
Genotyping primer for *Snca*^{G51D} reverse: anneal at 63 degree<br>5’- GTCATGCACCACGCCCTCTTTCGTCTTTG-3’	Sigma-Aldrich	N/A
Genotyping primer for WT sequence of *Snca*^{G51D} reverse: anneal at 63 degree<br>5’- TCCATGAACCACTCCTTCCTTAGTTTTGG-3’	Sigma-Aldrich	N/A
Genomic sequencing primer for *Snca*^{A30P} forward:<br>5’- GGAGCAAAAATACATCTTTAGCCA-3’	Sigma-Aldrich	N/A
Genomic sequencing primer for *Snca*^{A30P} reverse:<br>5’- GTTTGTTTCTGAGAGCTAATATGC-3’	Sigma-Aldrich	N/A
Genomic sequencing primer for *Snca*^{E46K}, *Snca*^{G51D} forward:<br>5’- GGTGGAACAGCACAGGAG-3’	Sigma-Aldrich	N/A
Genomic sequencing primer for *Snca*^{E46K}, *Snca*^{G51D} reverse:<br>5’- GGTCCATTGGTGATACCTGTAC-3’	Sigma-Aldrich	N/A
qPCR primer for *mSnca* forward:	Sigma-Aldrich	N/A

5’-GTGACAACAGTGGCTGAGAAGAC-3’		
qPCR primer for *mSnca* reverse: 5’-GGTACCCCTCCTCACCCTTG-3’	Sigma-Aldrich	N/A
qPCR primer for *mGapdh* forward: 5’-AGGTCGGTGTGAACGGATTTG-3’	Sigma-Aldrich	N/A
qPCR primer for *mGapdh* reverse: 5’-TGTAGACCATGTAGTTGAGGTCA-3’	Sigma-Aldrich	N/A

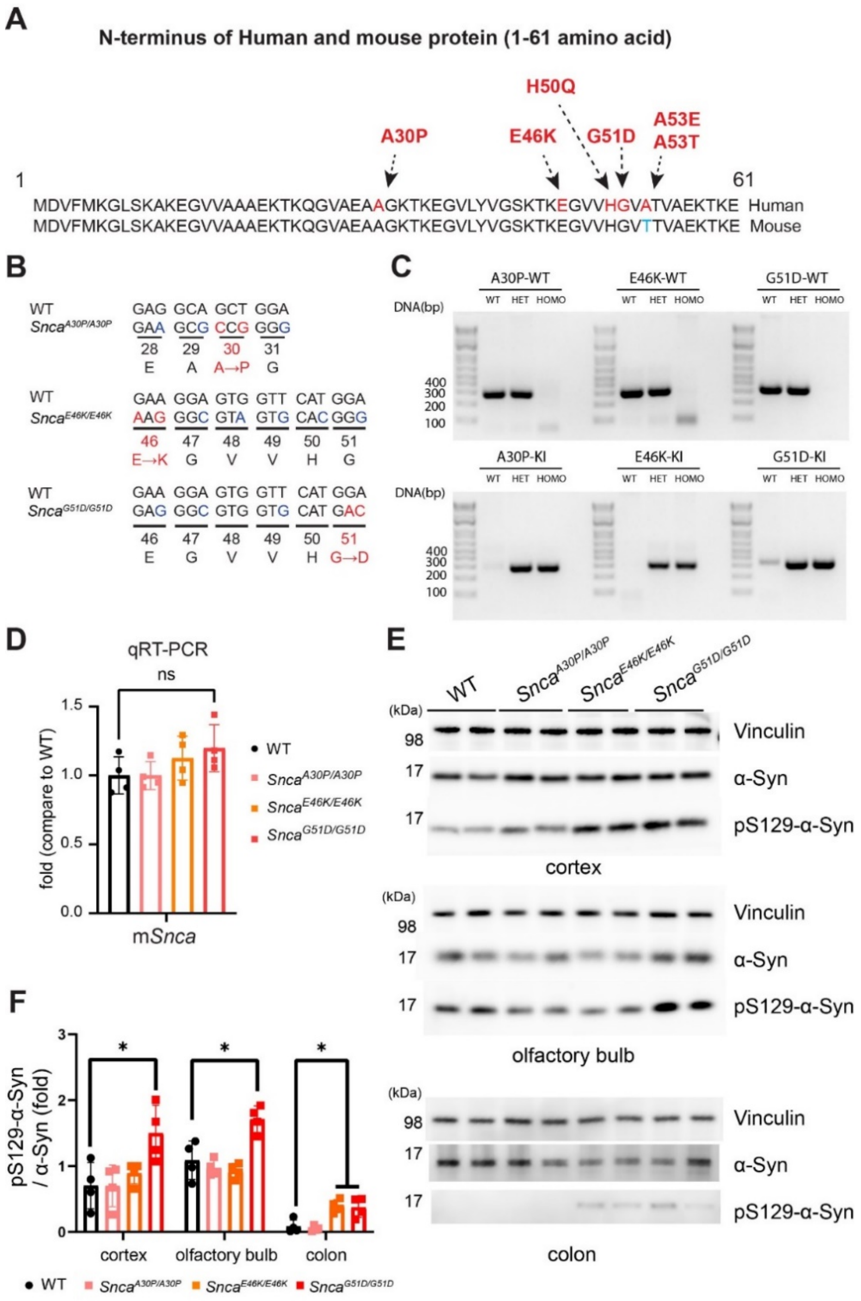

Figure S1. Generation of α-Synuclein familial mutant KI mice and assessment of α-Syn and pS129-α-Syn levels. (**A**) Known inherited mutations of SNCA that cause early-onset PD. Blue: known inherited mutations that cause early-onset PD for which a mouse model already exists. (**B**) Sequencing result of amino acid substitution from original to disease-associated mutations. Blue: synonymous mutation for genotyping, red: missense mutation that changes the amino acid to PD-causing mutation. (**C**) representative gel image for genotyping PCR for each KI mice. *Snca*$^{A30P/A30P}$ =280bp, *Snca*$^{E46K/E46K}$ and *Snca*$^{G51D/G51D}$ =320bp. (**D**) qRT-PCR data for mouse *Snca* levels in WT, *Snca*$^{A30P/A30P}$, *Snca*$^{E46K/E46K}$ and *Snca*$^{G51D/G51D}$ mice. n=4, n.s., not significant. (**E**) Immunoblot image of protein extract from the cortex, olfactory bulb, and colon of 3-month-old WT, *Snca*$^{A30P/A30P}$, *Snca*$^{E46K/E46K,}$ and *Snca*$^{G51D/G51D}$ mice. (**F**) The relative signal intensity of the blot of phospho-S129-α-Syn of WT, *Snca*$^{A30P/A30P}$, *Snca*$^{E46K/E46K,}$ and *Snca*$^{G51D/G51D}$ compared with WT total α-Synuclein. n=4, p*<0.05, ns, not significant.

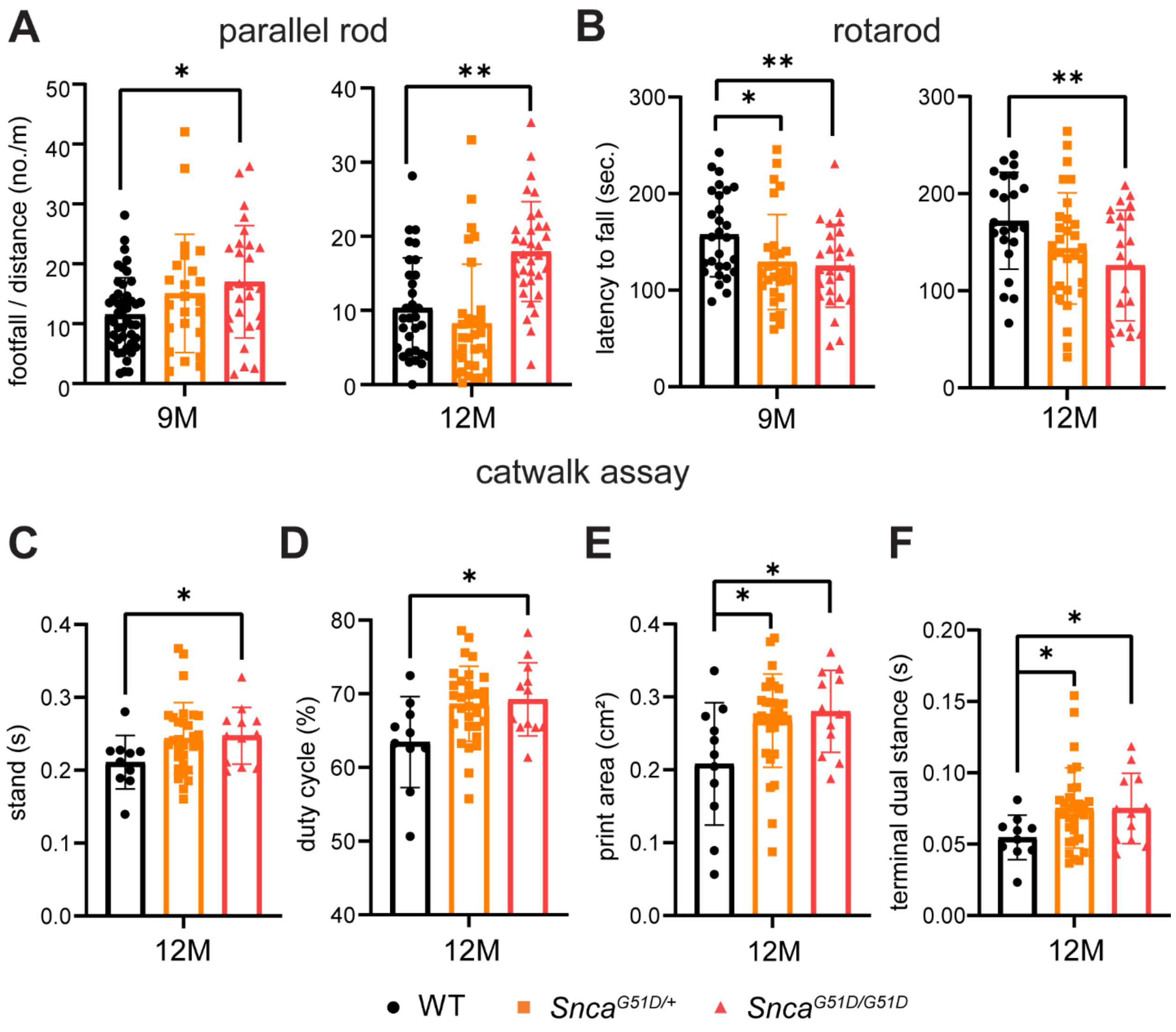

Figure S2. Homozygous G51D mice display greater motor incoordination than WT and heterozygous G51D mice. (**A**) Footslips relative to distance traveled on the parallel rod floor. WT (n=33, n=17 male, n=16 female), *Snca*$^{G51D/+}$ (n=30, n=15 male, n=15 female) and *Snca*$^{G51D/G51D}$ (n=29, n=15 male, n=14 female). (**B**) Latency to fall from the rotating rod. WT control (n=22, , n=11 male, n=11 female), *Snca*$^{G51D/+}$ (n=30, n=15 male, n=15 female) and *Snca*$^{G51D/G51D}$ (n=23, , n=12 male, n=11 female) WT and *Snca*$^{G51D/G51D}$ data reused from Figure 2A. (**C-F**) G51D mice show altered gait on the catwalk assay. WT control (n=10, n=5 male, n=5 female), *Snca*$^{G51D/+}$ (n=30, n=15 male, n=15 female) and *Snca*$^{G51D/G51D}$ (n=14, n=7 male, n=7 female). (**C**) stand, (**D**) duty cycle, (**E**) average printed area, and (**F**) terminal dual stance of the hind limbs. * $p<0.05$, ** $p<0.01$. WT and *Snca*$^{G51D/G51D}$ data were reused from Figure 1F and Figure 2.

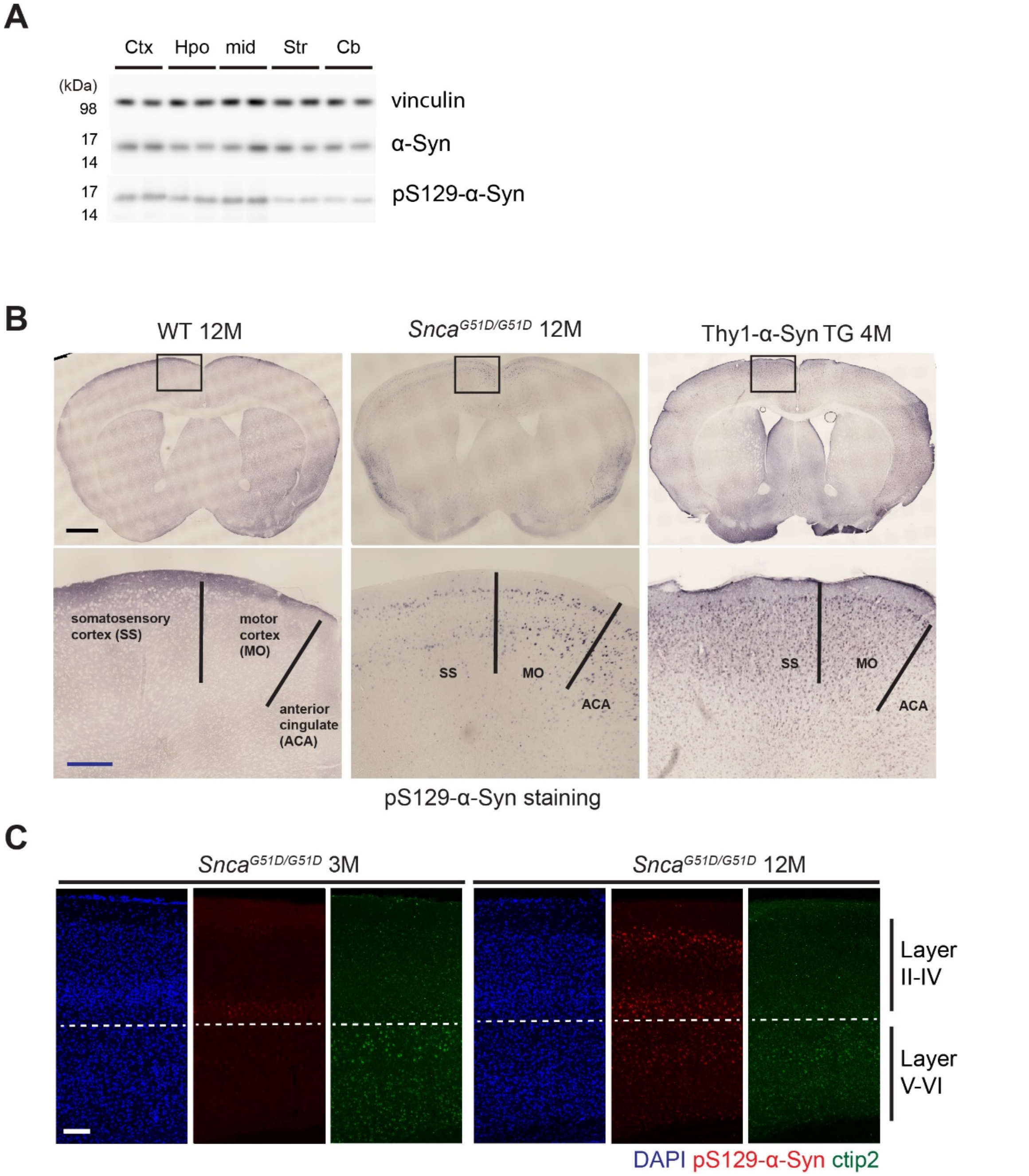

Figure S3. Phosphorylated α-Syn found in the whole brain and layers of the cortex of G51D mice. (**A**) Immunoblot image of protein extracts from the cortex, hippocampus, midbrain, striatum, and cerebellum of 12-month-old *Snca*$^{G51D/G51D}$ mice. (**B**) Phosphorylated pS129-α-Syn in 12-month-old WT controls, *Snca*$^{G51D/G51D}$ mice, and 4-month-old Thy1-α-Syn transgenic mice (line 61,). *Snca*$^{G51D/G51D}$ mice have pS129-α-Syn-positive cells in two different layers in the cortex, but Thy1-α-Syn transgenic mice show it in all layers of the cortex, even at an earlier age. Scale bars; black: 1mm, blue: 200µm. (**C**) Layer specification accumulation of phosphorylated α-Syn in the cortex based on Ctip2 expression. pS129-α-Syn expression is detected in layers II and IV at 3 months (upper panel) and spreads to layer V at 12 months (lower panel). Scale bar: 100µm.

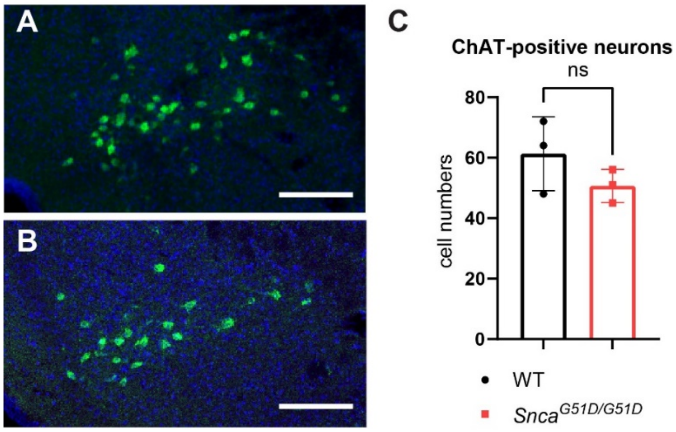

Figure S4. Cholinergic neurons in DMV neurons were not decreased in G51D mice. (**A-B**) Representative image of cholinergic neuron in DMV by acetyltransferase (ChAT) positive neurons (green) from 12-month WT (**A**) and G51D KI (**B**) mice. Scale bar: 50μm. (**C**) Quantification of ChAT-positive cells. No significant difference between WT and G51D mice. n=3.

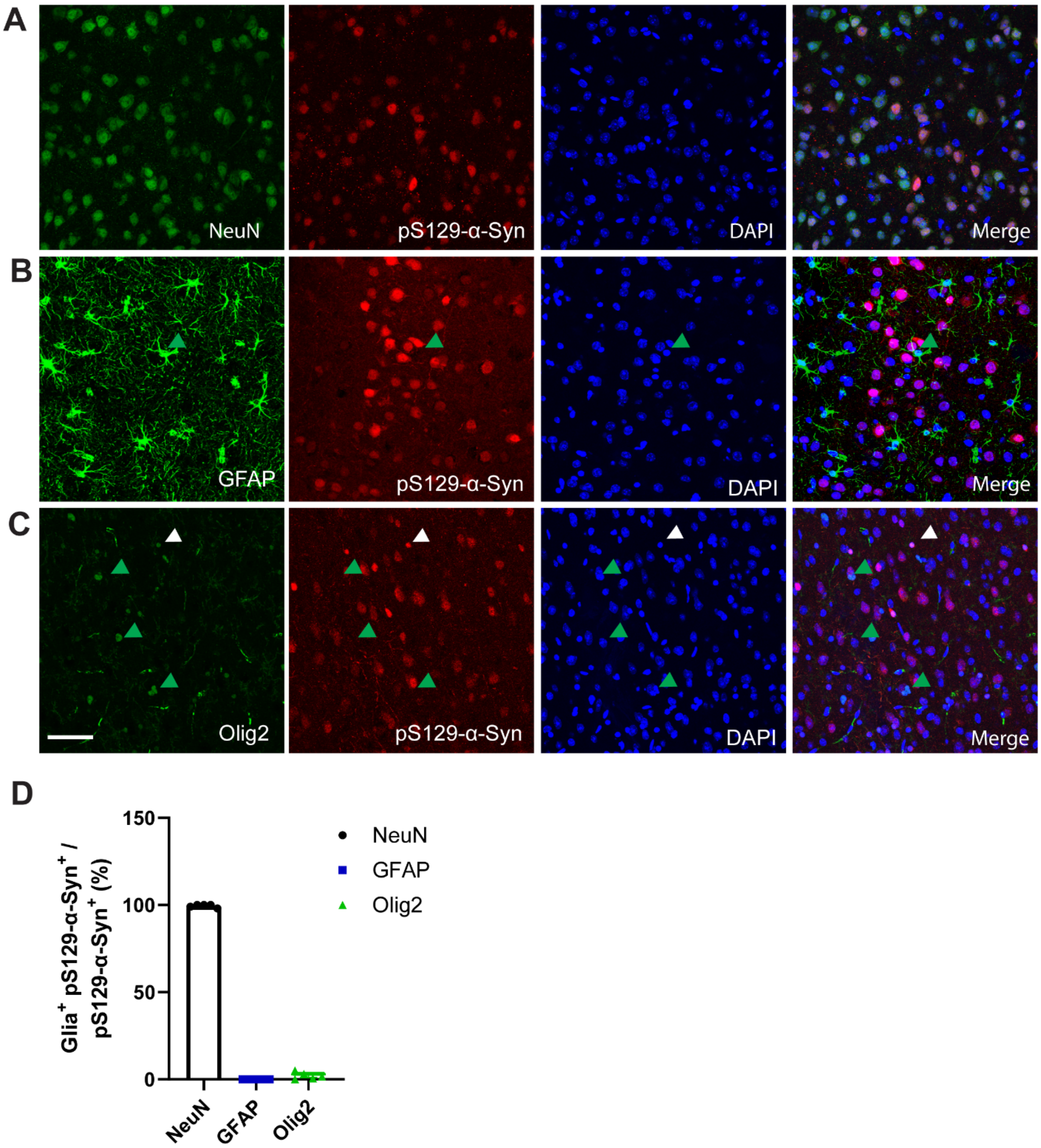

Figure S5. Phosphorylated α-syn at S129 is found mostly in neurons and some oligodendrocytes, but not astrocytes, of G51D mice. Representative images of co-staining of pS129-α-Syn with neuron or glial markers in 12-month-old *Snca*$^{G51D/G51D}$ cortices. Scale bar (white): 50µm. (**A**) pS129-α-Syn co-localized with neurons, co-stained with neuronal marker, NeuN. (**B**) pS129-α-Syn did not co-localize with active astrocytes (stained witgh GFAP). Green arrowhead: cells have GFAP but no p-α-Syn. (**C**) pS129-α-Syn rarely co-localized with oligodendrocytes (stained with Olig2). White arrowhead: cells co-localized with both Olig2 and pS129-α-Syn. Green arrowhead: cells have Olig2 but no pS129-α-Syn. (**D**) Quantification of pS129-α-Syn-positive cells with NeuN, GFAP and Olig2. Less than 5% of Olig2-positive oligodendrocytes colocalized with pS129-α-Syn. n=6.

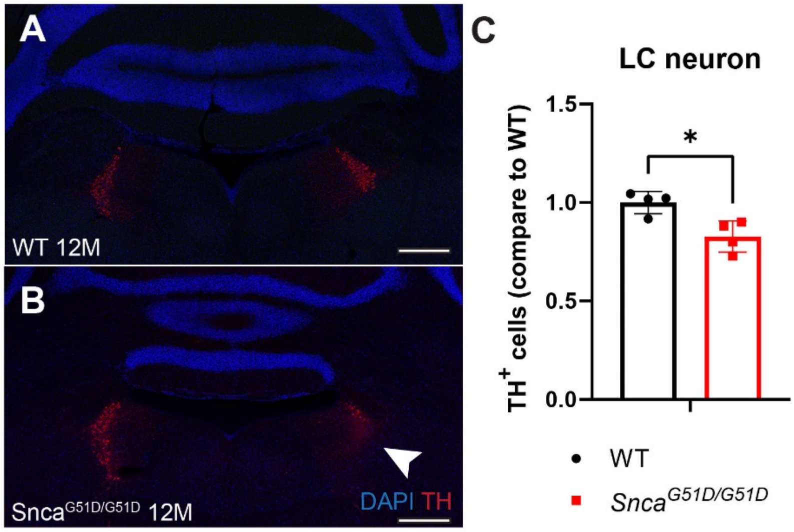

Figure S6. Loss of locus coeruleus neurons in G51D mice. (**A-B**) Representative image of TH-positive dopaminergic neurons in locus coeruleus of 12-month-old WT control (A) and G51D mice (B). Scale bar: 400µm. (**C**) Relative numbers of TH-positive cells in substantia nigra of 12-month-old WT control and mutant mice. n=4 for each, p*<0.05.

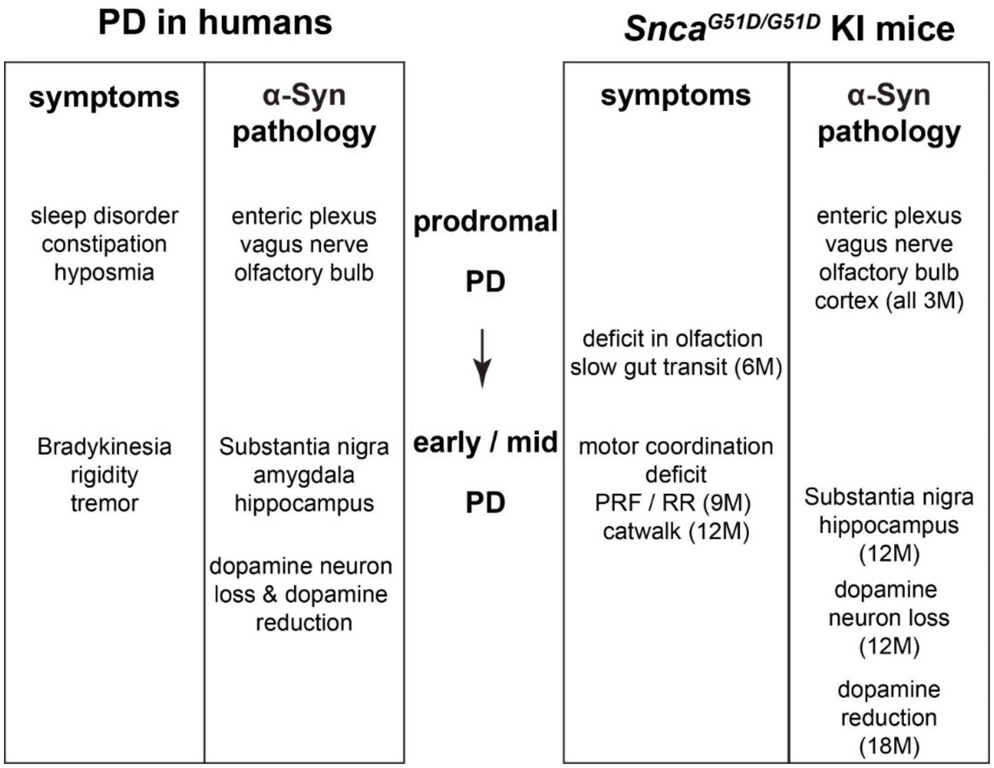

Figure S7. Parallels between human and G51D mouse pathology and symptoms.

Supplement information Reference

1. C. S. Chuang *et al.*, Quantitative evaluation of motor function before and after engraftment of dopaminergic neurons in a rat model of Parkinson's disease. *J Biomed Sci* **17**, 9 (2010).
2. J. Kim *et al.*, Evolutionarily conserved regulators of tau identify targets for new therapies. *Neuron* **111**, 824-838.e827 (2023).
3. J. Schindelin *et al.*, Fiji: an open-source platform for biological-image analysis. *Nat Methods* **9**, 676-682 (2012).
